# Revealing the Effect of Local Connectivity of Rigid Phases during Deformation at High Temperature of Cast AlSi12Cu4Ni(2,3)Mg Alloys

**DOI:** 10.3390/ma11081300

**Published:** 2018-07-27

**Authors:** Katrin Bugelnig, Holger Germann, Thomas Steffens, Federico Sket, Jérôme Adrien, Eric Maire, Elodie Boller, Guillermo Requena

**Affiliations:** 1 Institute of Materials Science and Technology, Technical University of Vienna, 13/308 Karlsplatz, A-1040 Vienna, Austria; 2 German Aerospace Centre, Linder Höhe, 51147 Cologne, Germany; guillermo.requena@dlr.de; 3 KS Kolbenschmidt GmbH, Karl-Schmidt-Straße, 74172 Neckarsulm, Germany; Holger.Germann@de.rheinmetall.com (H.G.); Thomas.Steffens@de.rheinmetall.com (T.S.); 4 IMDEA Materials Institute, C/Eric Kandel 2, 28906 Getafe, Spain; federico.sket@imdea.org; 5 Laboratoire MATEIS, UMR5510 CNRS, INSA Lyon, Université de Lyon, 69621 Villeurbanne, France; jerome.adrien@insa-lyon.fr (J.A.); eric.maire@insa-lyon.fr (E.M.); 6 ESRF—The European Synchrotron, CS40220 Grenoble CEDEX 9, France; boller@esrf.fr; 7 Metallic Structures and Materials Systems for Aerospace Engineering, RWTH Aachen University, 52062 Aachen, Germany

**Keywords:** Cast Al-Si alloys, 3D characterization, synchrotron tomography, in-situ tensile deformation, 3D microstructure, damage, strength, connectivity

## Abstract

The 3D microstructure and its effect on damage formation and accumulation during tensile deformation at 300 °C for cast, near eutectic AlSi12Cu4Ni2Mg and AlSi12Cu4Ni3Mg alloys has been investigated using in-situ synchrotron micro-tomography, complemented by conventional 2D characterization methods. An increase of Ni from 2 to 3 wt.% leads to a higher *local connectivity*, quantified by the Euler number *χ*, at constant *global interconnectivity* of rigid 3D networks formed by primary and eutectic Si and intermetallics owing to the formation of the plate-like Al-Ni-Cu-rich δ-phase. Damage initiates as micro-cracks through primary Si particles agglomerated in clusters and as voids at matrix/rigid phase interfaces. Coalescence of voids leads to final fracture with the main crack propagating along damaged rigid particles as well as through the matrix. The lower local connectivity of the rigid 3D network in the alloy with 2 wt.% Ni permits localized plastification of the matrix and helps accommodating more damage resulting in an increase of ductility with respect to AlSi12Cu4Ni3Mg. A simple load partition approach that considers the evolution of local connectivity of rigid networks as a function of strain is proposed based on in-situ experimental data.

## 1. Introduction

The strength of cast Al-Si piston alloys is given by the strength of the age-hardenable α-Al matrix and the load carrying capability of interconnected 3D hybrid networks formed by Si and various intermetallic phases [1,2,3,4,5]. The alloying elements Cu and Mg play a decisive role on precipitation strengthening of the matrix, while, together with Ni, they define the interconnectivity of the 3D networks [6,7,8,9]. These rigid networks exhibit high thermal stability and provide strength retention up to about 300 °C even after overaging of the matrix [10,11].

In recent years, the influence of the interconnectivity of the 3D networks on strength at ambient and elevated temperatures has been made evident by several investigations [1,11,12,13,14,15]. It is now well established that these networks have complex morphologies and it is therefore necessary to describe them considering their connectivity as a whole structure, termed here global interconnectivity [2], as well as connections within the network, that is, their local connectivity [16,17]. Thus, the ambient and elevated temperature strengths of Al-Si piston alloys are improved in the presence of ~20 vol.% of highly interconnected (>95% global interconnectivity) networks consisting of eutectic Si and Ni-, Cu-, Fe-rich intermetallics owing to a transfer of mechanical load from the weaker matrix to these rigid networks [1,11,12,13,14,18,19]. It is also well known now that Ni plays a decisive role in terms of enhancement of strength of these alloys, especially at high temperatures up to 300 °C [6,18,19,20,21]. An addition of at least 1 wt.% of Ni to a cast AlSi12 alloy resulted in the formation of 3D networks with a high contiguity between Si and aluminides and this reduced the spheroidisation of eutectic Si during solution treatment [11,12]. Moreover, an increase of Ni content from 1 to 2 wt.% in an AlSi10Cu5NiX was observed to result in an increase of room temperature strength by ~15% after 4 h solution treatment at 500 °C [1,15]. The reason for this is that, although in the as cast condition similar degrees of global interconnectivity of the rigid phases (Si + aluminides) are obtained, the addition of more than 1 wt.% Ni is necessary to avoid the partial disintegration of the 3D network during solution treatment.

It is however insufficient to consider only the global interconnectivity of these rigid 3D networks to understand the mechanical behavior of Al-Si piston alloys. We have recently shown that the room temperature tensile strength of an AlSi12Cu4Ni2 alloy [16] decreases after 4 h solution treatment at 500 °C although the strength of the α-Al matrix and the global interconnectivity of rigid networks remain constant. A closer look using synchrotron micro-tomography with µm resolution revealed that changes in the local connectivity of the networks occur during solution treatment as a result of partial dissolution of Al_2_Cu aluminides and preliminary states of Al_2_Cu (e.g., θ’, θ”) as well as slight spheroidisation and fragmentation of eutectic and primary Si particles. These changes were quantified using the topological parameter Euler number *χ* [17,22,23,24] and it was suggested that a decrease of local connectivity owing to the loss of connecting branches within the 3D network provoked a decrease in the load bearing capability of the globally fully interconnected 3D networks. The Euler number *χ* has been used to study theoretically the effect of local connectivity on strength of a periodic 2D microstructure by Silva et al. [24]. For this purpose, they randomly removed connections in their model material and found that a loss of 10% of connections resulted in a decrease of strength of about 35%. Moreover, Kruglova et al. [17] theoretically correlated an increase in strength of an AlSi7 alloy to a more negative Euler number of the 3D network formed by eutectic Si, that is, higher local connectivity of the load bearing phase. Thus, our experimental results at room temperature [16] and these theoretical studies clearly indicate that local connectivity should be taken into account to understand the mechanical behavior of these alloys. However, it must also be considered that the 3D rigid networks undergo damage during deformation and, therefore, both their global and local interconnectivities may gradually change. This means that the load carrying capability of the networks changes as well gradually as deformation advances.

In the current work, we present an experimental approach to quantify the evolution of local connectivity of rigid 3D networks as a function of damage during tensile deformation at 300 °C of AlSi12Cu4NixMg piston alloys (x = 2–3) using in-situ synchrotron X-ray computed tomography. This is to the best of our knowledge the first study that comprises quantification of the evolution of interconnectivity of 3D networks in Al-Si alloys experimentally and the introduction of a simple analytical load partition model that considers local connectivity changes as a function of strain. 

## 2. Materials and Methods

### 2.1. Materials

Two cast near-eutectic Al-Si piston alloys with the chemical compositions given in Table 1 were investigated.

**Figure 1:**
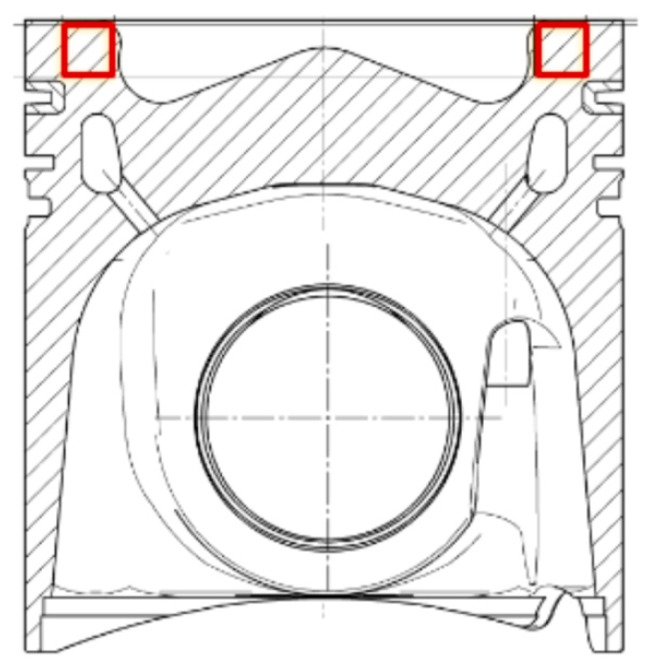
Illustration of the cross-section of a piston with the location of sample extraction at the bowl rim area indicated with red squares.

Piston raw parts produced by gravity die casting were manufactured by KS Kolbenschmidt GmbH, Neckarsulm, Germany. All the samples studied in this work were taken from the bowl rim area of these pistons (see Figure 1). Both alloys were subjected to ageing at 230 °C for 5 h followed by air cooling.

### 2.2. Methods

Light optical microscopy (LOM) and scanning electron microscopy (SEM) were complemented by synchrotron X-ray computed tomography (sXCT). sXCT can provide the necessary phase contrast to reveal simultaneously the α-Al matrix as well as eutectic and primary Si. This is not possible by laboratory XCT, owing to the very similar X-ray attenuations of these phases [2]. For a qualitative investigation of the interconnectivity of rigid phases, specimens were chemically etched using a H_2_O + HCl solution with a ratio of 60:40 for approximately 45 min to ensure a slow and gentle dissolution of the α-Al matrix without damage of the rigid phases or distortion of the hybrid network. Thereafter, scanning electron microscopy of the etched specimens was conducted and complemented with energy-dispersive X-ray spectroscopy (EDX).

The hardness of the alloys and of the α-Al matrix was determined by Brinell hardness HB (1/10) and nano-indentation, respectively. To obtain statistically relevant values, at least 5 Brinell hardness measurements were conducted on the sample surface of each specimen, while for the nano-hardness H of the Al matrix, indentations in at least 30 positions (3 groups with 10 indentations each) for each alloy were carried out. A detailed description of the experimental conditions applied for nano-indentation can be found in [16].

In-situ tensile tests were conducted at the beamline ID19 of the European Synchrotron Radiation Facility (ESRF) in Grenoble, France [25], using a tensile rig with elevated temperature capabilities provided by INSA Lyon, Lyon, France. Flat dog-bone shaped tensile samples with a total length of 40 mm and 1 mm^2^ cross-section at the gauge length of 2 mm were produced by spark erosion. Tensile tests were conducted at 300 °C at a strain rate of 1 µm/s and with controlled heating of the specimen central section by an induction coil monitored by a thermocouple glued to the sample. The test temperature was reached with a transient of a few seconds and was kept constant for the duration of the tensile test, with a maximum deviation of ±1 °C. Table 2 shows the experimental parameters for the sXCT scans carried out during the in-situ tensile tests at 300 °C. The first tomography was acquired before deformation and several tomographic scans were then subsequently acquired after applying increasing deformation steps until fracture. The determination of load steps of interest and the methodology for calculation of the global strain in the investigated volumes is described in detail in [16].

The 3D microstructure of the alloys in the initial condition was also studied by sXCT using a higher spatial resolution than for the in-situ tensile tests. For this purpose, cylindrical specimens with diameters of 0.6 mm were machined. An exposure time of 0.02 s/projection and 4999 projections per scan were acquired with a voxel size of 0.3^3^ µm^3^. 

### 2.3. Image Analysis

#### 2.3.1. Pre-Processing

Reconstruction of tomographic scans was carried out with a filtered backprojection algorithm. The volumes at different load steps of the tensile tests were registered using the rigid registration tool available in Avizo Fire 9.3 (Thermo Scientific, Waltham, MA, USA). Prior to segmentation, the reconstructed volumes were filtered using a 2D or 3D anisotropic diffusion filter available in Fiji [26] and Avizo Fire 9.3.

#### 2.3.2. Image Segmentation

Segmentation of aluminides was carried out using three different global grey value thresholds (best threshold determined by eye ±2 grey values) to increase the representativity of the quantitative analysis. An automatic segmentation of Si particles over a large volume was not possible by simple global grey value thresholding and, therefore, manual segmentation was carried out. 3D visualizations of regions of interest were produced using the software Avizo Fire.

#### 2.3.3. Characterization of the 3D Microstructure and Damage

The quantification of the 3D microstructure of the alloys in the initial condition was determined in volumes of ~420 × 400 × 1180 µm^3^. The interconnectivity of a phase, understood here as the global interconnectivity of that phase, was quantified as the volume of the largest particle divided by the total volume of all particles of the same phase in the studied volume, as defined in previous works [1,27]. Local connectivity, that is, connecting branches within a network, was quantified using the topological parameter Euler number χ [17,22,23,24]. A detailed explanation of the calculation of these parameters can be found in [16]. 

The morphology of primary Si was quantified aiming at identifying clusters formed by connected primary Si particles. For this, the aspect ratio and the sphericity, ψ, of primary Si particles were calculated as follows:

(1) $$\mathrm{aspect}\mathrm{ratio}=\frac{\mathrm{max.Feret}-\mathrm{diameter}}{\mathrm{min.Feret}-\mathrm{diameter}},\;$$

(2) Ψ=6π1/2vparticlesparticle32,Ψ∈[0,1],

where *V_particle_* is the particle volume in (µm^3^) and *S_particle_* is the particle surface in (µm^2^). A sphericity of 1 indicates spherical particles. A decrease in sphericity is accompanied by increasingly irregular particle shapes. On the other hand, an increase in aspect ratio indicates more elongated shapes.

For the characterization of damage during tensile deformation, a 3D Despeckle-filter (1 voxel), available in Avizo Fire, was applied to reduce artefacts after voids segmentation by global grey value thresholding. The smallest particle/void size considered for both the static and in-situ scans was 36 µm^3^. 

## 3. Results

### 3.1. Influence of Chemical Composition on the Microstructure

#### Initial Microstructure

**Figure 2:**
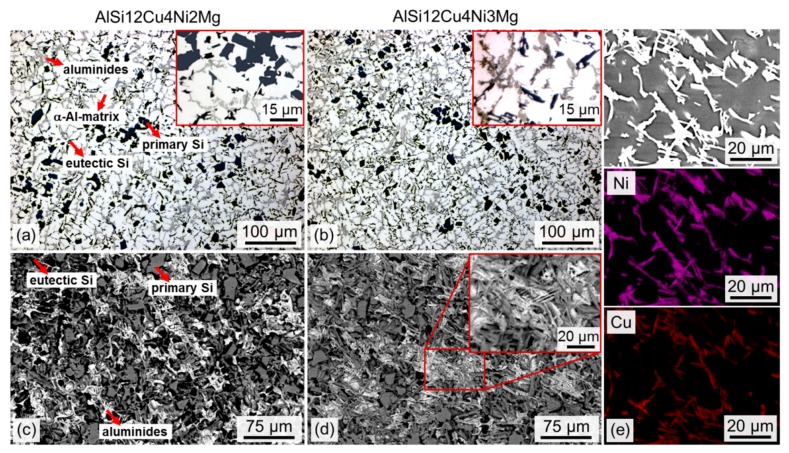
(**a**,**b**) light optical micrographs; (**c**,**d**) scanning electron microscopy (SEM) micrograph of the networks formed by Si and intermetallics after deep etching the α-Al matrix for AlSi12Cu4Ni2Mg (**a**,**c**) and AlSi12Cu4Ni3Mg (**b**,**d**); (**e**) SEM and energy-dispersive X-ray spectroscopy (EDX) maps of Ni and Cu for the AlSi12Cu4Ni3Mg alloy.

Figure 2a,b shows the 2D microstructure of the investigated alloys. Both alloys show the presence of dendrites with eutectic/primary Si and intermetallic phases (aluminides) in the interdendritic region. Moreover, clusters of connected primary Si particles result in a rather heterogeneous distribution of this phase. There are also several intermetallic phases containing Si, Cu, Ni, Mg, Mn and Fe [20,28,29]. SEM micrographs of deep etched specimens reveal a large fraction of needle-like intermetallics in the 3 wt.% Ni alloy (see insert in Figure 2d). EDX mappings reveal that these phases correspond to Al-Cu-Ni-rich aluminides (Figure 2e), i.e., most probably δ-phase (Al_3_CuNi) according to the literature (e.g., [29,30]).

Table 3 presents average values for Brinell hardness of the alloys (HB) and nano-hardness of the α-Al matrix (H). Although the results are very similar considering the experimental scatter, the AlSi12Cu4Ni3Mg alloy may have a slightly higher Brinell hardness than AlSi12Cu4Ni2Mg.

**Figure 3:**
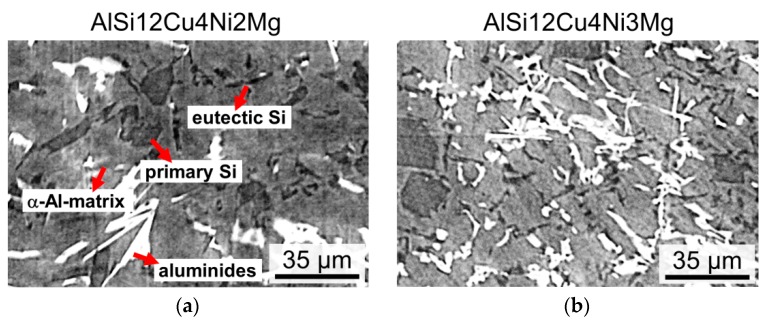
Portion of reconstructed synchrotron tomography slices: (**a**) AlSi12Cu4Ni2Mg and (**b**) AlSi12Cu4Ni3Mg.

Figure 3 displays portions of reconstructed sXCT slices of both alloys. Bright particles correspond to aluminides, while dark grey regions represent bulky primary Si particles and platelet-like eutectic Si.

**Figure 4:**
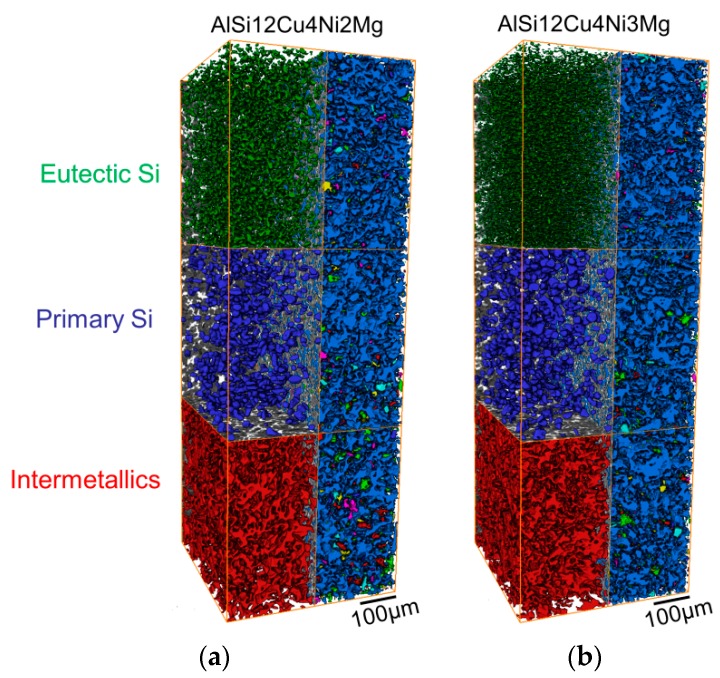
3D visualization of the networks formed by the rigid phases in (**a**) AlSi12Cu4Ni2Mg and (**b**) AlSi12Cu4Ni3Mg. Left half of each figure: red = intermetallics, blue = primary Si particles, green = eutectic Si. Right half: network form considering all rigid phases together. Investigated volume: ~420 × 400 × 1180 µm^3^.

Figure 4 shows visualizations of the 3D networks formed by intermetallics and eutectic + primary Si in a volume of 420 × 400 × 1180 µm^3^. The left half of the visualization shows each segmented microstructural component separately: red = intermetallics, blue = primary Si particles, green = eutectic Si. On the other hand, the right halves of Figure 4a,b show all the rigid phases embedded in the transparent α-Al-matrix. Different colors have been assigned to each individual particle in this half of the volumes. While the aluminides (intermetallics) as well as primary + eutectic Si form globally highly interconnected 3D networks on their own (left half of each figure), all these phases together form a globally practically fully connected network in both alloys (right half of each figure) [1,16].

**Figure 5:**
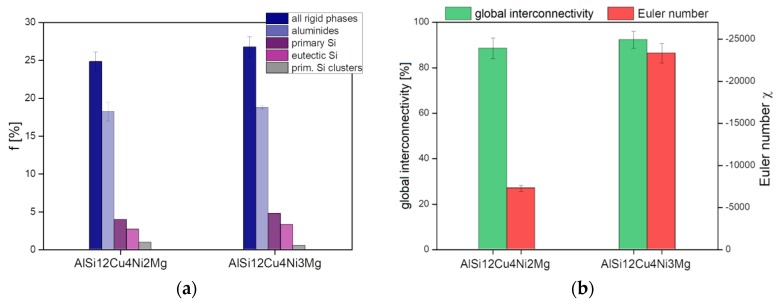
(**a**) Volume Fraction (*f*) of rigid phases, (**b**) global interconnectivity and Euler number *χ*, of the 3D network considering all rigid phases.

The volume fraction, *f*, global interconnectivity and Euler number, *χ*, of Si and aluminides before deformation determined in volumes of 420 × 400 × 1180 µm^3^ are shown in Figure 5a,b individually and combined, that is, for the hybrid 3D network shown in Figure 4. Both alloys possess similar volume fractions of rigid phases (see Figure 5a) and—from a global point of view—highly interconnected networks with global interconnectivities of about 95% (see Figure 5b, green bars). A considerable difference between the 3D hybrid networks of the two alloys is given by their *local connectivity*, quantified here using the topological parameter Euler number *χ* (Figure 5b, red bars). The lowest Euler number (−23,326 ± −1115) for the 3D network of AlSi12Cu4Ni3Mg indicates the highest local connectivity, that is, a larger number of connecting branches within the 3D network than for the alloy with 2 wt.% Ni, which shows about 41% less local connectivity (χ = −7277 ± −436). 

**Figure 6:**
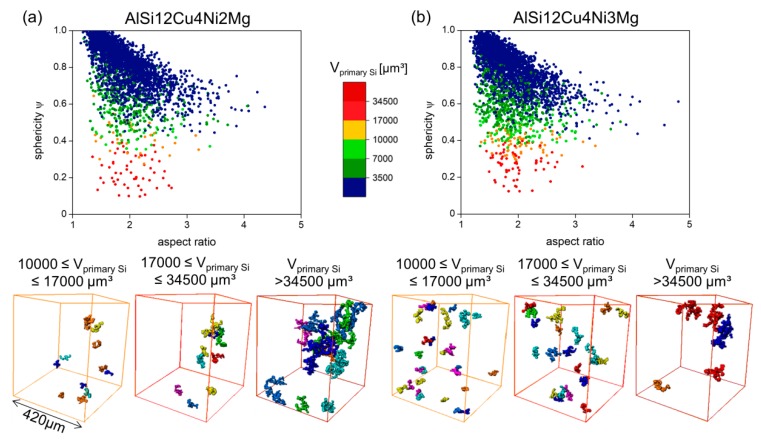
Sphericity-Aspect ratio-Volume diagrams for primary Si: (**a**) AlSi12Cu4Ni2Mg and (**b**) AlSi12Cu4Ni3Mg. The bottom of the figure shows representative 3D visualization of primary Si clusters for 10,000 µm^3^ ≤ V_primary Si_ ≤ 17,000 µm^3^ (yellow), 17,000 µm^3^ ≤ V_primary Si_ ≤ 34,500 µm^3^ (red) and V_primary Si_ > 34,500 µm^3^ (brown).

Large primary Si particles have frequently been reported to be favorable damage initiation sites during tensile deformation (e.g., [31,32,33,34]), however, the crucial effect of primary Si clusters on damage of near eutectic Al-Si alloys has received much less attention [35,36], thus, a detailed investigation of primary Si particles has been conducted. Figure 6 shows quantitatively the relationship between sphericity, aspect ratio and size of primary Si particles in the form of scatter diagrams. Color categories for several particle size ranges have been defined based on morphological changes observed qualitatively: (i) individual primary Si particles can be found for V_primary Si_ ≤ 3500 µm^3^; (ii) small clusters of primary Si particles are present within 3500 µm^3^ ≤ V_primary Si_ ≤ 7000 µm^3^); (iii) larger clusters with up to about 15 connected particles are within 7000 µm^3^ ≤ V_primary Si_ ≤ 10,000 µm^3^; and (iv) very large, irregular clusters formed by more than 15 connected particles are found for V_primary Si_ ≥ 10,000 µm^3^. Representative 3D visualizations of primary Si clusters for the size range(iv) are shown at the bottom of Figure 6 for the 2 wt.% Ni alloy (three images at the left hand side) and the 3 wt.% Ni alloy (3 images at the right hand side).

The sphericity evolves from rather spherical shapes at smaller particle sizes towards very irregular shapes with increasing size for both alloys. Clusters > 34,500 µm^3^ are heterogeneously distributed in the microstructure of both investigated alloys. The volume fraction of primary Si in the investigated volumes is 3.9 vol.% and 4.7 vol.% for the 2 wt.% Ni and the 3 wt.% Ni alloy, respectively. The larger volume fraction of primary Si can be attributed to the slightly higher Si content in the 3 wt.% Ni alloy (see Table 1). However, the volume fraction of the largest primary Si clusters in the investigated volumes (V_primary Si_ > 34,500 µm^3^) amounts to ~0.9 vol.% and ~0.5 vol.% for the 2 wt.% and 3 wt.% Ni alloy, respectively.

### 3.2. Tensile Tests at 300 °C

**Figure 7:**
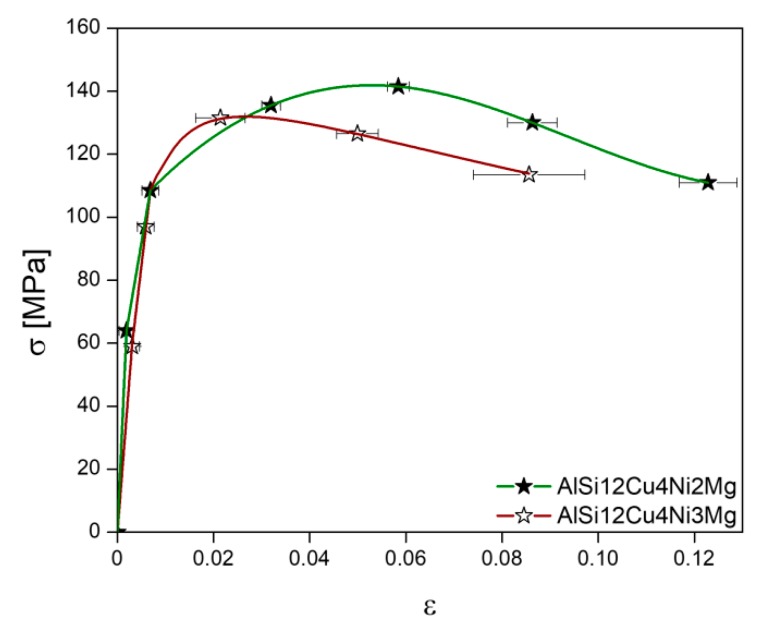
Stress-strain curves obtained during the in-situ tensile tests at 300 °C. The star-like symbols indicate the conditions at which synchrotron tomography was conducted.

Figure 7 shows the stress-strain curves acquired during the in-situ tensile tests at 300 °C. The star-like symbols indicate the conditions at which sXCT was conducted. Both alloys show similar stress-strain evolution up to about 110 MPa. The 2 wt.% Ni alloy shows the largest elongation at failure (ε = 0.12 ± 0.006) and also the highest ultimate tensile strength σ_UTS_ = 141 MPa occurring at ε = 0.058 ± 0.002 compared to the 3 wt.% Ni alloy, for which σ_UTS_ = 132 MPa is reached already at ε = 0.0215 ± 0.005 and the elongation at fracture is ε = 0.086 ± 0.01.

#### Damage Formation and Accumulation during Tensile Tests at 300 °C

**Figure 8:**
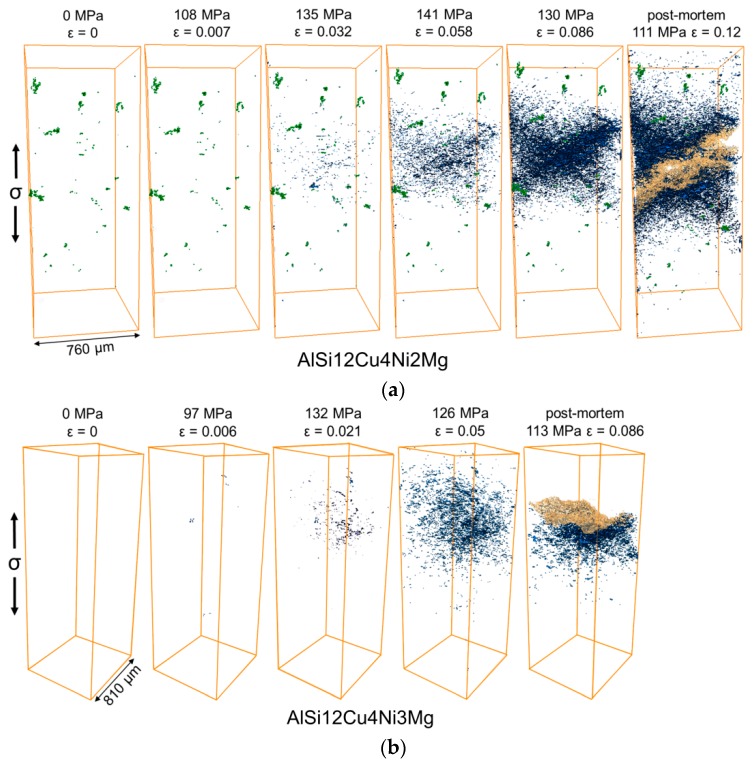
3D visualization of processing porosity (green), voids (blue) near the fracture surface (beige) at several load steps during the in-situ tensile tests at 300 °C: (**a**) AlSi12Cu4Ni2Mg; (**b**) AlSi12Cu4Ni3Mg. The load direction is vertical.

Figure 8a,b shows the damage (blue) at several deformation steps during tensile deformation at 300 °C as wells as in the post-mortem condition for the AlSi12Cu4Ni2Mg and AlSi12Cu4Ni3Mg alloys, respectively. The regions shown in yellow in the post-mortem conditions correspond to the fracture surface (the upper half of the fractured sample of the AlSi12Cu4Ni3Mg alloy could not be scanned and it is therefore missing in Figure 8b). It can also be seen qualitatively that AlSi12Cu4Ni2Mg shows a larger number and volume fraction of voids before final failure compared to AlSi12Cu4Ni3Mg, indicating that the alloy with less Ni is able to accommodate more damage before fracture. Damage forms and accumulates in a rather localized manner for both alloys. Moreover, AlSi12Cu4Ni2Mg shows the presence of randomly distributed processing porosity (see green particles in unloaded condition of this alloy in Figure 8a), which arises as a result of metal shrinkage during solidification. Damage was not observed to proceed from these shrinkage pores.

**Figure 9:**
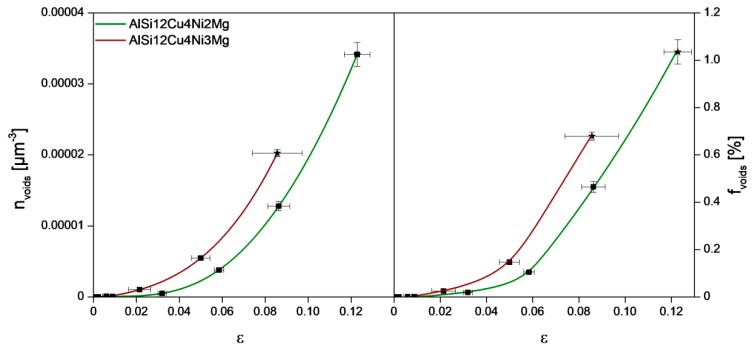
Number density (*n_voids_*) and volume fraction (*f_voids_*) of voids at several deformation steps.

The accumulation of damage is shown quantitatively in Figure 9 considering the number density (*n_voids_*) and volume fraction (*f_voids_*) of voids as a function of strain. Here, no distinction is made between micro-cracks and rounder voids. AlSi12Cu4Ni2Mg displays less damage than AlSi12Cu4Ni3Mg at the same applied strain and accumulates a larger fraction of damage prior to final failure.

**Figure 10:**
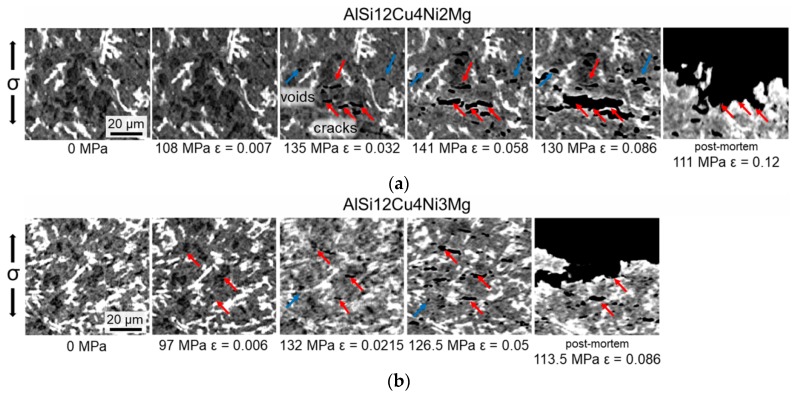
Damage mechanisms observed during the in-situ tensile tests: (**a**) AlSi12Cu4Ni2Mg; (**b**) AlSi12Cu4Ni3Mg. The load direction is vertical.

Figure 10 shows representative portions of tomographic slices where the dominant damage mechanisms leading to fracture can be observed. Although the same damage mechanisms were observed for both alloys, damage was detected at earlier stages for the alloy with 3 wt.% Ni, that is, at σ = 97 MPa and ε = 0.006 ± 0.002 for AlSi12Cu4Ni3Mg, compared to σ = 135 MPa and ε = 0.032 ± 0.002 for AlSi12Cu4Ni2Mg, revealing the capability of the latter alloy to accommodate a larger amount of plastic deformation before damage initiation. Damage begins in the form of micro-cracks through primary Si particles agglomerated in clusters (red arrows in Figure 10). Round voids at the interfaces between α-Al-Matrix and the rigid phases were observed as well simultaneously for the AlSi12Cu4MgNi2 alloy, while this mechanism is detected at later stages of deformation for the alloy with 3 wt.% Ni (blue arrows in Figure 10). While the formation of cracks at primary Si particles forming cluster was also observed in our previous investigations during in-situ tensile tests of similar alloys at room temperature [16], the decohesion between matrix and rigid phases takes place only at elevated temperature. Lebyodkin et al. [34] reported that this damage mechanism is more likely to occur at elevated temperatures owing to relaxation of stresses at the interface between Al and Si. The cracks through Si particles are oriented preferentially perpendicular to the load direction.

**Figure 11:**
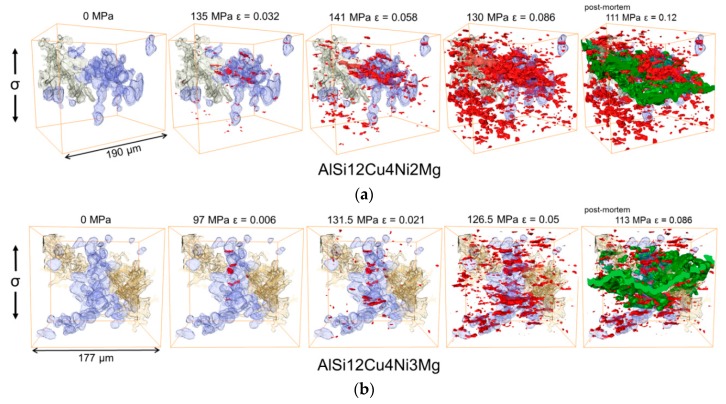
3D visualization of damage accumulation at several load steps and in the post-mortem condition (main crack = green): (**a**) AlSi12Cu4Ni2Mg alloy and (**b**) AlSi12Cu4Ni3Mg alloy. Damage is shown in red, while primary Si particles are blue and aluminides beige. The matrix is transparent.

Figure 11 shows 3D visualizations of the damage mechanisms during deformation for volumes containing the 2D slices shown in Figure 10. Only the primary Si particles (blue) and an aluminide particle connected to the Si cluster are shown in this figure. Eutectic Si and further aluminides which are also present within this volume were made transparent to improve clarity. The micro-cracks through primary Si particles observed in the 2D slices result in a fragmentation of the originally fully connected particle cluster (blue) causing a localized damage accumulation in the early stages of failure. Furthermore, these 3D visualizations reveal that isolated Si particles can break if they are located close to clusters. It is also clear from these volumes that voids can also be formed in the α-Al-matrix, which is another distinction with respect to the damage mechanisms observed at room temperature (RT) [16] (some of the voids formed in the matrix while a fraction of them is located in eutectic Si and aluminides—or at their interface—transparent in this figure). Final failure occurs by coalescence of micro-cracks and voids, regardless of the alloy. Contrary to RT observations [16], where the main crack propagates exclusively along connecting micro-cracks formed at Si and aluminides, at 300 °C, propagation through voids in the α-Al-matrix is also observed (see the green crack, part of the main crack, shown in Figure 11). Even though damage mechanisms are the same for both alloys, the damage onset strain differs clearly between the alloys, as it can be seen in Figure 10 and Figure 11. 

**Figure 12:**
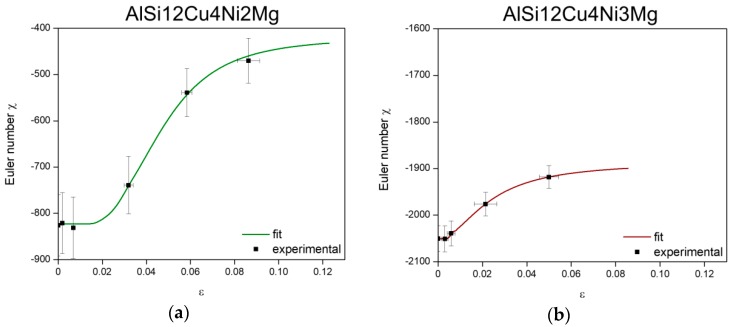
Evolution of Euler number of the 3D rigid network during tensile deformation for the volumes shown in Figure 11: (**a**) AlSi12Cu4Ni2Mg and (**b**) AlSi12Cu4Ni3Mg.

The local connectivity of the 3D networks (i.e., Euler number χ) changes as damage accumulates since this results in their fragmentation. Figure 12 shows the evolution of the Euler number of the 3D networks at several load steps calculated in the volumes shown in Figure 11 considering the full network of rigid phases contained in these volumes.

The Euler number remains constant until damage initiates. Then, an increase of χ with progressing deformation takes place owing to the loss of connecting branches by the formation of micro-cracks that progressively disintegrate the network. This increase of χ is more significant for the 2 wt.% Ni alloy (from initially −826 to −470 at the step before failure) in comparison to the 3 wt.% Ni alloy (from initially −2050 to −1886 at the step before failure). The experimentally determined evolution of the Euler number of the network as a function of strain, *χ*_n_(ε), was fitted with an expression of the form:

(3) $$\chi_{n}\left(\varepsilon \right)=A_{2}+\frac{A_{1}-A_{2}}{\left(1+{\left(\frac{\varepsilon }{x_{0}}\right)}^{p}\right)},$$

where A_1_ is the initial value, A_2_ is the final value, p is the power and x_0_ is the center of the curve. The fitting parameters for each alloy are given in Table 4.

## 4. Discussion

### 4.1. Influence of Chemical Composition on the Microstructure and Damage Evolution

The sXCT results show that primary Si clusters play a major role in damage formation during tensile deformation at 300 °C, as it has also been observed at room temperature during a previous in-situ study of the tensile behavior of a similar alloy [16]. Both alloys studied in this work display large clusters of primary Si (V_primary Si_ ≥ 34,500 µm^3^, see Figure 6). It is known that Cu and Mg enhance the formation of dendrites by extending the solidification range of the alloys, which, in turn, allows the formation of primary Si chain-like clusters in the interdendritic space [6,29,37].

The damage mechanisms and their sequence identified during the in-situ tensile tests at 300 °C are very similar for both alloys:

1. Formation of micro-cracks through primary Si particles agglomerated in clusters, voids at matrix/rigid phase interfaces as well as voids in the matrix can be seen in the early stages of damage. Fracture of isolated primary Si particles and eutectic Si can also occur if they are located in the vicinity of primary Si clusters. While the formation of voids in the matrix and at the interface between the matrix and the rigid phases was observed simultaneously with micro-cracking of primary Si in the AlSi12Cu4Ni2Mg alloy, these two mechanisms seem to occur at a later deformation stage for AlSi12Cu4Ni3Mg. Further sXCT scans at strains between 0.007 and 0.0032 are necessary for the AlSi12Cu4Ni2Mg alloy to fully clarify this difference.
2. Coalescence of voids leading to final failure with the main crack propagating along damaged rigid particles as well as through the matrix.

This is in agreement with previous reports in which an increase in the fraction of decohesion between Si/matrix interfaces and matrix damage in shape of voids was also observed after deformation of Al-Si alloys at elevated temperature [33,34,38,39,40]. Decohesion is known to result from accumulation of plastic deformation of the matrix in the vicinity of rigid phases, while fracture of rigid particles occurs owing to incompatible stresses between rigid phase and matrix [33]. Elevated temperatures facilitate plastic deformation of the matrix, and thus aid in alleviating the mismatch stresses, promoting decohesion instead of fracture [34]. Furthermore, a transition from brittle to ductile fracture modes with increasing test-temperature owing to crack propagation through the matrix has frequently been detected (e.g., [38,39]).

On the other hand, damage forms at earlier deformation steps for the 3% Ni alloy, which can be observed in Figure 10 and Figure 11. The higher Ni content, results in the formation of plate-like δ-phase (see Figure 2b,d), resulting in the lowest Euler number (−23,326 ± −1115) and thus, the highest local connectivity, for the 3D network of AlSi12Cu4Ni3Mg (see Figure 5b). The earlier damage initiation can be attributed to the increase of rigidity of the 3D network, restricting the plastic deformation of the matrix. The 3D network in AlSi12Cu4Ni2Mg has about 41% less local connectivity (χ = −7277 ± −436) compared to the 3 wt.% Ni alloy, that is, a considerably lower amount of connecting branches. This permits some plastic deformation of the matrix and helps accommodating more damage resulting in an increase of ductility with respect to AlSi12Cu4Ni3Mg, while the presence of more connecting branches in AlSi12Cu4Ni3Mg favors crack propagation through the rigid network and, consequently, final fracture occurs at lower strains with respect to the 2 wt.% Ni alloy. 

### 4.2. Analytical Stress Partition Model to Gain Further Insights into the Mechanical Behavior of Al-Si Piston Alloys

It can be argued that a higher local connectivity increases the load carrying capability of the 3D networks, that is, more branches locally reinforcing the structure. Thus, a low Euler number (more negative) enhances the load transfer from the matrix to the rigid 3D network reducing the portion of load that must be borne by the matrix. In a first attempt to rationalize the effect of local connectivity on the load partition between matrix and the Si + aluminides 3D network, it is plausible to propose:

(4) $$\;\frac{\sigma_{m}}{\sigma_{n}}=-\frac{C}{\chi },\;$$

where σ_m_ is the load carried by the α-Al matrix, σ_n_ is the load carried by the rigid 3D network, C is a constant and χ is the Euler number. To determine C one can consider a condition at which deformation of the alloy is in the linear regime and damage has not yet initiated, e.g., the first deformation conditions for which tomography was carried out (see Figure 7). Here, the alloys can be assumed as continuously reinforced composites owing to the high *global interconnectivity* of the 3D networks, hence the matrix and the 3D network experience the same deformation:

(5) $$\varepsilon_{0}=\varepsilon_{n0}=\varepsilon_{m0},$$

where ε_n0_ is the strain in the network and ε_m0_ is the strain in the matrix at ε_0_. Approximating with the Hook’s law:

(6) $$\varepsilon_{0}=\frac{\sigma_{m0}}{E_{m}}=\frac{\sigma_{n0}}{E_{n}},$$

E_m_ can be approximated to the Young’s Modulus of AA6061 at 300 °C (0.66 wt.% Si, 0.9 wt.% Mg, 0.24 wt.% Cu) [41]:

(7) $$E_{m}=50\;\mathrm{GPa},$$

Deriving from that, the matrix stress at the first deformation condition measured by sXCT results in $\sigma_{m0}=\sim \;52\;\mathrm{MPa}$, based on the acquired stress-strain curves (Figure 7) with a practically identical elastic regime for both alloys. 

The stress in the network at ε_0_ can then be calculated as:

(8) $$\sigma_{n0}=\frac{E_{n}\times \sigma_{m0}}{E_{m}},$$

E_n_ can be approximated as:

(9) $$E_{n}=f_{\mathrm{Si}}\times E_{\mathrm{Si}}+f_{\mathrm{aluminides}}\times {\bar{E}}_{\mathrm{aluminides}},$$

where f_Si_ is 0.066 and 0.081 while f_aluminides_ is 0.18 and 0.19 for the 2 wt.% Ni and the 3 wt.% Ni alloy, respectively (see Figure 5a). Several intermetallic phases must be considered to determine the Young’s Modulus of the aluminides network $\bar{E}$_aluminides_, therefore it is approximated here as the average of the Young’s moduli measured at 200 °C for each present intermetallic phase taken from [42] (since the difference in Young’s Modulus between these phases does not differ much—(± 6 GPa)—we assume the same fraction of phases for the calculation of the average Young’s Modules $\bar{E}$_aluminides_):

(10) $${\bar{E}}_{\mathrm{aluminides}}=144\;\mathrm{GPa},$$

The Young’s Modulus of Si (E_Si_) in dependence of temperature can be given in form of a linear equation approximated based on experimental data acquired in [42] in a temperature range 200 °C ≥ T ≤ 350 °C,

(11) $$E_{\mathrm{Si}}\;\left(T\right)\;=\;125.9\;–\;0.07333\;\times \;T,$$

According to this equation E_Si_ at 300 °C was estimated as,

(12) $$E_{\mathrm{Si}}\left(300\;{}^{\circ}C\right)=104\;\mathrm{GPa},$$

Using these values, E_n_ = 33 GPa and 36 GPa for the networks in AlSi12Cu4Ni2Mg and AlSi12Cu4Ni3Mg, respectively. With this, σ_n0_ = 36 MPa and 39 MPa for the 2 wt.% Ni alloy and the 3 wt.% Ni alloy, respectively. As a result, we can calculate C for each alloy,

(13) $$C_{2\%}=-1251\;\mathrm{Pa},$$

(14) $$C_{3\%}=-3184\;\mathrm{Pa},$$

The stress partition between the matrix and the 3D network can also be approximated by a simple rule of mixtures since, as previously mentioned, the high interconnectivity of the network permits an analogy with a continuously reinforced material:

(15) $$\sigma =f_{n}\times \sigma_{n}+f_{m}\times \sigma_{m}=f_{n}\times \sigma_{n}+\left(1-f_{n}\right)\times \sigma_{m},$$

where f_m_ is the matrix volume fraction and f_n_ the volume fraction of the 3D network (Si + aluminides).

As mentioned in the results section, the local connectivity of the 3D networks, i.e., the Euler number, continuously changes during deformation owing to the formation and accumulation of damage. The evolution of Euler number as a function of applied strain, $\chi_{n}\left(\varepsilon \right)$, (see Figure 12) is used to implement the effect of local connectivity changes into the stress partition model. Combining Equations (3), (4) and (15) we can calculate the stress borne by the network as damage advances as:

(16) $$\sigma_{n}=\sigma \times \frac{1}{\left(f_{n}+\frac{C}{\chi_{n}\left(\varepsilon \right)}\times \left(f_{n}-1\right)\right)},$$

**Figure 13:**
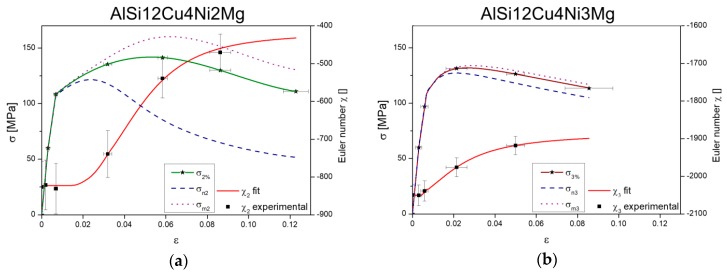
Evolution of stresses in the network and matrix calculated taking into account the disintegration of the 3D rigid networks by damage accumulation: (**a**) AlSi12Cu4Ni2Mg and (**b**) AlSi12Cu4Ni3Mg.

The global stress σ is taken from the experimentally acquired stress-strain curves in Figure 7. The results of this approximation are plotted in Figure 13a,b for both alloys together with the evolution of χ of the 3D rigid networks and stress-strain curves from the in-situ tensile tests. The subscripts 2 and 3 are used for the 2 wt.% Ni and the 3 wt.% Ni alloy, respectively.

In the linear regime the network and matrix show the same evolution as the global stress-strain curves for both alloys. As damage begins, reflected by the increase of χ, the evolution of load borne by the matrices and the networks start to diverge. A more significant decrease of $\sigma_{n}$ can be observed for the 2 wt.% Ni alloy after reaching a maximum at ~120 MPa, while, complementarily, $\sigma_{m2}$ further increases by strain hardening up to ~160 MPa. On the other hand, $\sigma_{n3}$ and $\sigma_{m3}$ follow a very similar trend throughout the tensile test since damage is less pronounced and, thus, the local connectivity of the 3D network is less affected than for the alloy with 2 wt.% Ni. Moreover, the load carried by the 3D network of AlSi12Cu4Ni3Mg is always higher than for the alloy with less Ni in the non-linear regime, revealing the higher reinforcing capability of the hybrid network owing to a higher local connectivity. However, the high rigidity of the 3D network in the 3 wt.% Ni alloy hinders plastic deformation of the α-Al matrix and the possibility to further increase the strength of the alloy by strain hardening as deformation proceeds. $\sigma_{n3}$ remains high and cracks in the network tend to coalesce and lead to earlier failure of the alloy with respect to the alloy with less Ni. Contrarily, the lower connectivity of the 3D network in AlSi12Cu4Ni2Mg has two consequences: first, its lower rigidity permits some plastic deformation and strain hardening of the α-Al matrix resulting in the higher strength of this alloy with respect to the alloy with 3 wt.% Ni and, second, more damage can be accommodated (in the matrix and at the interface between the matrix and the 3D network) leading to higher elongation at fracture than for AlSi12Cu4Ni3Mg. 

This simple model, that takes into account the evolution of local connectivity of the 3D rigid networks as damage accumulates in cast Al-Si piston alloys, sheds a light on the effect of damage on strength of these materials and it is, to the best knowledge of the authors, the first attempt to rationalize the effect of global interconnectivity and local connectivity analytically using actual data obtained in-situ, during deformation at high temperature.

## 5. Conclusions

The influence of the 3D microstructure on the damage evolution of AlSi12Cu4Ni2Mg and AlSi12Cu4Ni3Mg cast piston alloys was studied during tensile deformation at 300 °C. The following conclusions are drawn:

- The additional formation of platelet-like Al-,Cu-,Ni-rich δ-phase owing to the increase of Ni content by 1 wt.% results in a larger amount of connecting branches and thus, a significant increase of *local connectivity* (quantified by the Euler number *χ*) of the rigid 3D network at practically constant *global interconnectivity* for the 3 wt.% Ni alloy.
- The in-situ tensile tests at 300 °C revealed ~10% higher strength and ~30% higher elongation at fracture of the 2 wt.% Ni alloy as compared to the 3 wt.% Ni alloy. Damage mechanisms during tensile deformation are the same for both alloys:
  ○ Damage mainly initiates as micro-cracking through primary Si particles agglomerated in clusters, voids at matrix/rigid phase interfaces, as well as voids in the matrix. Moreover, isolated primary Si particles and eutectic Si can break if they are located close to clusters. Failure occurs by coalescence of voids with the main crack propagating along fractured rigid particles as well as through the α-Al-matrix.
  ○ The lower local connectivity of the 3D network in AlSi12Cu4Ni2Mg permits local plastification of the matrix and helps accommodating more damage resulting in an increase of ductility with respect to AlSi12Cu4Ni3Mg. On the other hand, the 3 wt.% Ni alloy reveals damage onset at earlier deformation steps and less damage accumulation until failure compared to the 2% Ni alloy. The presence of more connecting branches in AlSi12Cu4Ni3Mg favors crack propagation through the rigid network and, consequently, final fracture occurs at lower strains with respect to the 2 wt.% Ni alloy.
- The evolution of local connectivity of the rigid 3D networks with damage accumulation based on experimental observations was implemented in a simple analytical stress partition model. The lower local connectivity at practically constant global interconnectivity of the 3D network in AlSi12Cu4Ni2Mg indicates two consequences:
  ○ its lower rigidity allows local plastification and strain hardening of the α-Al matrix resulting in the higher strength of this alloy.
  ○ more damage can be accommodated leading to higher elongation at fracture than for AlSi12Cu4Ni3Mg.

## Figures and Tables

**Table 1:** Chemical compositions of the investigated alloys (wt.%).

Alloy	Al	Si	Cu	Ni	Mg
AlSi12Cu4Ni2Mg	bal.	12.5	4	2	1
AlSi12Cu4Ni3Mg	bal.	13.1	4	3	1

**Table 2:** Experimental parameters for tomography during the in-situ tensile tests at the beamline ID19/ European Synchrotron Radiation Facility (ESRF).

Experiment	Detector	Energy (keV)	FOV (mm^2^)	Sample to Detector Distance (mm)	Exposure Time (s/proj)	Proj.	Voxel Size (µm^3^)	Total Scan Time (s)
In-situ tensile tests at 300 °C	PCO Dimax	19	1 × 2	150	0.01	1000	1.1^3^	20

**Table 3:** Brinell-hardness of the alloys and nano-hardness of the α-Al matrix.

Alloy	HB (1/10)	H (GPa)
AlSi12Cu4Ni2Mg	126 ± 2.7	1.8 ± 0.1
AlSi12Cu4Ni3Mg	129 ± 1.4	1.8 ± 0.1

**Table 4:** Fit-parameters for Euler number curves in Figure 12.

Parameter	AlSi12Cu4Ni2Mg	AlSi12Cu4Ni3Mg
A_1_	−822.5	−2051.4
A_2_	−420.2	−1886.5
p	3.6	1.9
x_0_	0.047	0.023
